# Role of Ldb1 in the transition from chromatin looping to transcription activation

**DOI:** 10.1186/1756-8935-6-S1-P41

**Published:** 2013-03-18

**Authors:** Ivan Krivega, Ann Dean

**Affiliations:** 1 Laboratory of Cellular and Developmental Biology, NIDDK, NIH, Bethesda, MD, 20892, USA

## Background

Long-range interaction between the beta-globin LCR enhancer and gene involves a multi-protein complex that includes Ldb1, LMO2, GATA-1 and TAL1 (Ldb1 complex). Ldb1 is required for long-range chromatin looping and beta-globin gene activation. Ldb1 can homodimerize *in vitro* through its N-terminal domain (DD). We tested whether the dimerization domain of Ldb1 plays a key role in LCR/beta-globin looping and transcription activation in erythroid cells.

## Materials and methods

We analyzed the ability of different deletions and fusions of Ldb1 to rescue beta-globin transcription in Ldb1 KD MEL cells. Chromatin immunoprecipitation, co-immunoprecipitation and 3C assays were used to identify protein occupancy at chromatin, interaction between proteins and the level of long-range interaction between cis-acting DNA elements respectively.

## Results

Co-immunoprecipitation experiments using wild type MEL cells expressing the Ldb1 DD confirmed that the DD homodimerizes with endogenous Ldb1 and, through it, interacts with other members of the Ldb1 complex. Full-length shRNA-immune Ldb1 rescued long-range LCR/beta-globin interaction, Ldb1 complex and RNA polymerase II (pol II) occupancy at the beta-globin promoter and beta-globin gene expression in Ldb1 knock-down MEL cells. A fusion protein between DD and LMO2 (LMO-DD) behaved similarly in fully restoring the structure and function of the locus, confirming the sufficiency of the DD domain for the long-rage interaction and beta-globin expression. Ldb1 proteins with deletions of 2 short conserved sequences within the DD (Ldb1Δ1 and Ldb1Δ2) expressed in the background of Ldb1 KD MEL cells were unable to rescue long-range LCR/beta-globin interaction, Ldb1 complex and pol II occupancy at the promoter or beta-globin gene expression. Additionally Ldb1 proteins with these DD deletions were unable to homodimerize with endogenous Ldb1. Ldb1 protein with a different deletion of a short conserved sequence within the DD (Ldb1Δ4/5), close to its C-terminus, also was unable to rescue pol II occupancy or beta-globin gene expression but it rescued Ldb1 complex occupancy at the beta-globin promoter and the long-range LCR/beta-globin interaction. Additional chromatin immunoprecipitation experiments showed the necessity of the 4/5 region of the Ldb1 DD for SWI/SNF, NuRD and FOG1 occupancy at the beta-globin gene promoter. Co-immunoprecipitation experiments showed a direct interaction between Ldb1 and FOG1 through the 4/5 region of the DD.

## Conclusions

Homodimerization between Ldb1 proteins facilitates long-range interaction between the LCR enhancer and beta-globin gene. Our study of DD mutants of Ldb1 have revealed that looping between the LCR and beta-globin gene can be established without pol II or transcription of the gene. The 4/5 region of the Ldb1 is dispensable for Ldb1 dimerization but is required for transcription of beta-globin. Ldb1 interacts with FOG1 (*friend of GATA1*) through the DD 4/5 region and this interaction is required for SWI/SNF, NuRD complex and pol II occupancy at the beta-globin promoter.

